# Apoptotic Effects of Chrysin in Human Cancer Cell Lines

**DOI:** 10.3390/ijms11052188

**Published:** 2010-05-19

**Authors:** Boon Yin Khoo, Siang Ling Chua, Prabha Balaram

**Affiliations:** Institute for Research in Molecular Medicine (INFORMM), Universiti Sains Malaysia, 11800 USM, Penang, Malaysia; E-Mails: siang_ling85@yahoo.com (S.L.C.); prabha@kb.usm.my (P.B.)

**Keywords:** chrysin, apoptotic effect, human cancers, *in vitro*

## Abstract

Chrysin is a natural flavonoid currently under investigation due to its important biological anti-cancer properties. In most of the cancer cells tested, chrysin has shown to inhibit proliferation and induce apoptosis, and is more potent than other tested flavonoids in leukemia cells, where chrysin is likely to act via activation of caspases and inactivation of Akt signaling in the cells. Moreover, structure-activity relationships have revealed that the chemical structure of chrysin meets the key structural requirements of flavonoids for potent cytotoxicity in leukemia cells. It is possible that combination therapy or modified chrysin could be more potent than single-agent use or administration of unmodified chrysin. This study may help to develop ways of improving the effectiveness of chrysin in the treatment of leukemia and other human cancers *in vitro*.

## 1. Introduction of Flavonoids

**Figure 1.**
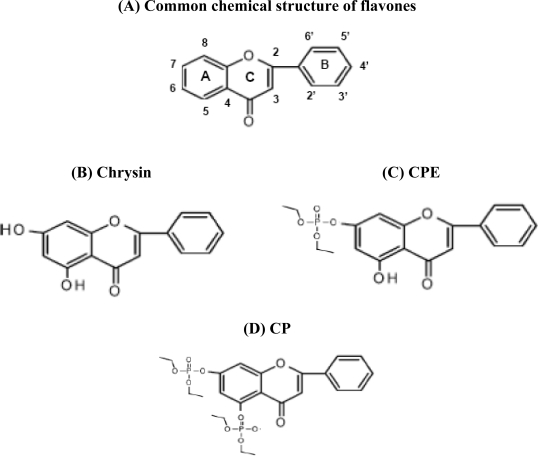
(**A**) Common chemical structure of flavones. Flavones have a common chemical structure consisting of fused A and C rings, and a phenyl B ring attached to position 2 of the C ring. (**B**) Chrysin is in the flavone subgroup of flavonoids and shares a common flavone structure with hydroxyls at position 5 and 7 of the A ring. Replacing the hydroxyl with a phosphate group at position 7, such as in diethyl chrysin-7-yl phosphate (CPE). (**C**) or at positions 5 and 7, such as in tetraethyl bis-phosphoric ester of chrysin (CP). (**D**), enhances the anti-cancer potential of the chrysin.

Flavonoids are a broad class of plant pigments that are ubiquitously present in fruit- and vegetable-derived foods [1,2]. Flavonoids can be easily ingested and a high level of flavonoids in food has been identified as an important constituent of the human diet. More than 4,000 types of biologically active flavonoids have been identified [3], which can be further divided into flavonols, flavones, flavanols, flavanones, anthocyanidins and isoflavonoid subclasses [4]. Chrysin, which is the focus of this review, is a flavone. The flavones have a common chemical structure, consisting of fused A and C rings, and a phenyl B ring attached to position 2 of the C ring (Figure 1A). Flavones, such as apigenin, baicalein, chrysin and scutellarein, were recently identified as having important biological roles in nitrogen fixation and chemical defenses [3–11].

Among the flavonoids studied, apigenin [5,7-dihydroxy-2-(4-hydroxyphenyl)-4*H*-chromen-4-one] has shown a remarkable inhibitory effect on cancer cell growth in both *in vitro* and *in vivo* tumor models [12,13]. Apigenin also possesses anti-inflammatory and free radical scavenging properties in several cancer cell lines [14,15], and inhibits tumor cell invasion, metastasis [16], mitogen-activated protein kinases (MAPKs) and its downstream oncogenes [17]. Chrysin (5,7-dihydroxy-2-phenyl-4H-chromen-4-one) is an analog of apigenin [18,19], but its anti-cancer properties have rarely been studied. Chrysin shares the common flavone structure with additional hydroxyls at positions 5 and 7 of the A ring (Figure 1B). Chrysin has recently shown to be a potent inhibitor of aromatase [18] and of human immunodeficiency virus activation in models of latent infection [20]. It has also demonstrated anti-inflammatory [21] and anti-oxidant effects [22], and has shown cancer chemopreventive activity via induction of apoptosis in diverse range of human and rat cell types. However, studies of the effects of chrysin on human cancers remain rare.

Activation of apoptosis is the key molecular mechanism responsible for the anti-cancer activities of most of the currently studied potential anti-cancer agents, including chrysin. Apoptosis contrasts with cell necrosis, in which the cells suffer a major insult, resulting in loss of membrane integrity, swelling and disruption [23]. During necrosis, the cellular contents are uncontrollably released into the extracellular environment, causing damage to surrounding cells and a strong inflammatory response in the corresponding tissues. In contrast, apoptosis induces cell shrinkage, chromatin condensation and margination at the nuclear periphery, with the eventual formation of membrane-bound apoptotic bodies containing organelles, cytosol and nuclear fragments, which are then phagocytosed without triggering inflammatory processes in the surrounding tissues. Although the chemical structure of chrysin with only two hydroxyls at position 5 and 7 of A ring showed lower cytotoxicity activity in certain human cancer cells, the potential apoptotic effect of chrysin has been reported in human cervical cancer, leukemia, esophageal squamous carcinoma, malignant glioma, breast carcinoma, prostate cancer, non-small cell lung cancer (NSCLC) and colon cancer *in vitro*, as outlined in Table 1.

## 2. Chrysin Inhibits Proliferation and Induces Apoptosis in HeLa Cells

A study by Zhang *et al*. [24] demonstrated that chrysin and its derivatives [diethyl chrysin-7-yl phosphate (CPE: C_19_H_19_O_7_P) and tetraethyl bis-phosphoric ester of chrysin (CP: C_23_H_28_O_10_P_2_)] exhibited potential anti-cancer effects in human cervical carcinoma. The chemical structures showed that CPE and CP have phosphate groups at positions 5 and/or 7 of the A ring, respectively (Figure 1C and 1D), which replace the hydroxyls at positions 5 and/or 7 of the A ring in chrysin. According to this study, chrysin and phosphorylated chrysin effectively inhibited the growth of cervical cancer cells, HeLa, via apoptosis induction and down-regulated the proliferating cell nuclear antigen (PCNA) in the cells. However, how the chrysin improved the resistant of TRAIL-induced apoptosis in HeLa cells was not mentioned in this study. Another study showed that chrysin potentially induced p38, therefore activated NFkappaB/p65 in the HeLa cells [25]. The MAPK p38 pathway has been implicated in the regulation of a wide spectrum of cellular processes, including cell-cycle arrest and apoptosis. Besides, it has been regarded as a potential phosphate donor for the p65 subunit of NFkappaB. According to the study [25], treatment of HeLa cells with 30 μM chrysin for 24 h induced a significant increase of NFkappaB/p65 levels in the cells, as demonstrated by EMSA. The signals could be suppressed by a specific p38 or p65 inhibitor indicating that the p38 or p65 could be useful therapeutic targets of chrysin to control gene expression in HeLa cells. However, no correlation of pro-apoptotic or apoptotic activity induced by chrysin in this phenomenon was clearly stated in the study. Although, chrysin was found to significantly sensitize the TNFalpha-induced apoptosis in human colorectal cancer cell line HCT-116, human liver cancer cell line HepG2, and the human nasopharyngeal carcinoma cell line CNE-1, in which such sensitization is closely associated with inhibitory effect on NFkappaB activation, the phenomenon may occur differently in HeLa cells [35]. Therefore, the NFkappaB remains a potential target to study the mechanism of apoptosis induced by chrysin in HeLa cells.

Although both chrysin and phosphorylated chrysin could inhibit proliferation and induced apoptosis in HeLa cells, as mentioned above, the effects of the phosphorylated chrysins were likely more potent than that of non-phosphorylated chrysin, where the estimated IC_50_ for chrysin was 14.2 μM, followed by CPE (IC_50_ = 10.3 μM) and CP (IC_50_ = 9.8 μM), assessed by the cell viability assays [24]. Phosphorylated chrysin(s), which could easily form non-covalent compound with lysozyme, are thus concluded as more effective in inhibiting cancer cell growth and inducing apoptosis than non-phosphorylated chrysin in HeLa cells.

## 3. Chrysin Induces Apoptosis in Leukemia Cells

In one study, 22 different flavonoids and related compounds were screened in human leukemia cells, U937. Among the flavonoids tested, genistein, apigenin, alpha-naphto-flavone, chrysin, quercetin, galangin, luteolin, fisetin and 3,7-dihydroxyflavone were found to significantly reduce the cellular viability of the U937 cells. However, only apigenin, chrysin, quercetin, galangin, luteolin and fisetin were found to clearly induce the oligonucleosomal DNA fragmentation at 50 μM after 6 h of treatment [26]. Chrysin was the most effective flavonoid in terms of reducing the viability of the U937 cells with an IC_50_ of 16 μM. Chrysin also potentiated the effects of TNFalpha in triggering apoptosis in the cells. On the other hand, Woo *et al*. [27] showed that chrysin induced apoptosis in association with activation of caspase-3, involving inactivation of Akt or Protein Kinases B (PKB) signaling and down-regulation of X-linked inhibitor of apoptosis protein (XIAP) in the U937 cells. This study provided the first evidence of a more detailed molecular mechanism whereby chrysin induces the apoptosis in leukemia cells namely via Akt dephosphorylation of the phosphoinositide 3 kinase (PI3K) signaling pathway.

**Figure 2.**
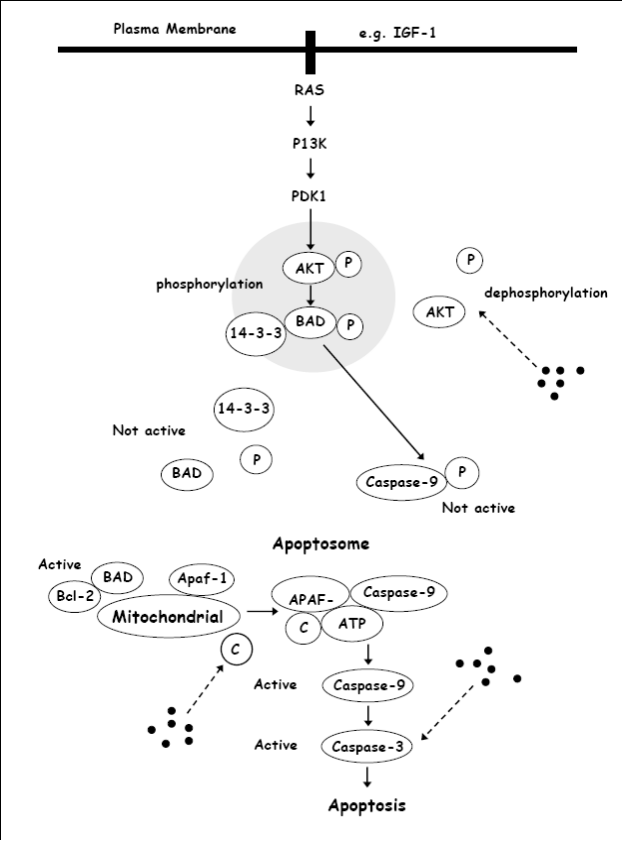
The PI3K/Akt signaling pathway. Chrysin is likely to act via activation of caspases and inactivation of Akt signaling in leukemia cells. (•) depicts chrysin.

The Akt signaling pathway, from PI3K to phosphoinositide-dependent kinase-1 (PDK1) and from PDK1 to Akt, mediates apoptosis in human cancer cells (Figure 2). Activation of Akt via phosphorylation prevents apoptosis [36], whereas dephosphorylation is likely to initiate apoptosis. Phosphorylation of Akt phosphorylates BAD (Bcl-2-associated death protein) and a non-active form of caspase-9, which are the hosts of the cell-signaling proteins. Phosphorylated BAD binds to cytosolic 14-3-3 proteins, resulting in a failure of the protein to heterodimerize with Bcl-2 at the mitochondrial membrane [37]. Dephosphorylation of BAD releases BAD from cytosolic 14-3-3 proteins, which subsequently form heterodimers with Bcl-2 family proteins and migrate into the mitochondrial membrane, where they induce the release of cytochrome *c* by altering the membrane pores [38,39]. Cytochrome *c* in the cytoplasm combines with Apaf-1 and caspase-9 to form a complex termed an apoptosome, in the presence of ATP, in order to activate the caspase-9 [39]. The caspase-9 subsequently activates the downstream executor caspase-3. Activation of caspase-3 and the subsequent degradative events probably trigger apoptosis [39,40]. Conversely, phosphorylation of caspase-9 by phosphorylated Akt prevents formation of the apoptosome complex, and therefore the downstream event of apoptosis is inhibited.

Woo *et al*. [27] noted several important effects of chrysin in U937 cells: (1) chrysin mediated the release of cytochrome *c* from mitochondria into the cytoplasm; (2) chrysin induced elevated caspase-3 activity and proteolytic cleavage of its downstream targets, such as phospholipase C-gamma-1 (PLC-gamma1), which is correlated with down-regulation of XIAP; and (3) chrysin decreased phosphorylated Akt levels in cells where the PI3K pathway plays a role in regulating the mechanism. These results suggested that chrysin-induced apoptosis was likely to be caspase- and mitochondria-dependent, and probably occurs via deregulation of PI3K/Akt, with involvement of XIAP. However, no measurement of BAD protein levels was reported in this study. The results of this study are in agreement with many other studies showing that chrysin, alone or in combination with other compounds, decreased the Akt phosphorylation and potentially resulted in mitochondrial dysfunction in leukemia cells [28,30]. Chrysin has also been reported to have the ability to abolish the stem cell factor (SCF)/c-Kit signaling by inhibiting the PI3K pathway [29]. In addition, Monasterio *et al*. [26] reported that flavonoids, including chrysin, induced apoptosis via a mechanism that required the activation of caspase-3 and caspase-8, indicating that chrysin-induced apoptosis could act via a ligand receptor-dependent cell death mechanism. This study also suggested a relationship between Akt and NFkappaB signaling in the cells. However, more studies are warranted to further evaluate the relationship of Akt and NFkappaB in the chrysin-treated leukemia cells.

A significant decrease in human telomerase reverse transcriptase (hTERT) expression levels was also observed in leukemia cells treated with 60 ng/mL *Manisa propolis*, owing to its constituent of chrysin [41]. Other studies, such as that of Josipovic and Orsolic [42], demonstrated that chrysin (as well as quercetin and caffeic acid) showed a high level of cytotoxicity in leukemia cells. The methanol extracts of apigenin, baicalein, chrysin, luteolin and wogonin have also shown a strong anti-leukemic activity [43]. All these studies indicated that chrysin exhibited potential anti-leukemic activities, suggesting its use as a potential anti-leukemic agent. The proliferation inhibitory effects of most of the flavonoids, including chrysin, in leukemia cells appear to be dose-dependent [44]. Moreover, structure-activity relationship studies reveal that the chemical structure of chrysin, which consists of a 2,3-double bond of C ring, a B ring attached to C ring at position 2, appropriate hydroxyls at position 5 and 7 of A ring, are likely to meet the key structural requirements of flavonoids for potent cytotoxicity in leukemia cells [26].

## 4. Cytotoxicity of Chrysin in Esophageal Squamous Carcinoma

The cytotoxic effects of structurally related flavones (luteolin, apigenin, chrysin) and flavonols (quercetin, kaempferol, myricetin), as well as the molecular mechanisms responsible for the cytotoxic effects in a human esophageal squamous cell carcinoma cell line, KYSE-510, have been determined by Zhang *et al*. [32]. The results of MTT assays showed that chrysin, as well as other flavonoids tested, were able to induce the cytotoxicity in KYSE-510 cells in dose- and time-dependent manners. Chrysin was estimated to have an IC_50_ of 63 μM in the cell line. Flow cytometry and DNA fragmentation analyses indicated that the cytotoxicity induced by chrysin (80 μM) and other flavonoids for 24 h was mediated by G(2)/M cell cycle arrest and apoptosis. Furthermore, the study revealed that treatment of KYSE-510 cells with chrysin (and other flavonoids) caused G(2)/M arrest through up-regulation of p21(waf1) and down-regulation of cyclin B1 at the mRNA and protein levels. In addition, the induction of apoptosis was p53-independent, but mitochondria-mediated through an up-regulation of p53-inducible gene 3 (PIG3) and cleavage of caspase-9 and caspase-3. The results of western blot analysis further showed that the increases in p63 and p73 translation or stability might contribute to the regulation of p21(waf1), cyclin B1 and PIG3 in the chrysin-induced KYSE-510 cells.

## 5. Chrysin in Malignant Glioma, Breast Carcinoma, Prostate and Other Human Cancers

In a study by Parajuli *et al*. [11], chrysin exhibited tumor-specific effects in diverse range of human cell lines, including malignant glioma cells (U87-MG and U-251), breast carcinoma cells (MDA-MB-231) and prostate cancer cells (PC3). Chrysin and other flavonoids (apigenin, baicalein, baicalin, scutellarein and wogonin) extracted from *Scutellaria* plants, showed dose-dependent inhibition of U87-MG proliferation. Apigenin was the most potent flavonoid, with IC_30_, IC_50_ and IC_70_ of approximately 16 μM, 62 μM and 250 μM, respectively, compared to IC_30_, IC_50_ and IC_70_ for chrysin of approximately 40 μM, 100 μM and 200 μM, respectively. This study also found that all six flavonoids, including chrysin (100 μM), significantly inhibited the proliferation of MDA-MB-231 cells, where a significant 43% inhibition was observed following treatment with chrysin. Chrysin also significantly inhibited the proliferation of U-251 and PC3 cells at 100 μM concentrations. All flavonoids examined, except scutellarein, also displayed significantly higher apoptotic activity in U87-MG cells compared to untreated U87-MG cells. The induction of apoptosis was significantly enhanced by increasing the dose of flavonoids, and further enhanced by prolonging treatment time from 72 h to 96 h. In this case, baicalein and baicalin produced the highest levels of apoptosis in U87-MG cells, followed by wogonin, apigenin, chrysin and scutellarein (not statistically significant), in accordance. However, the study did not report any details regarding the apoptotic activity of chrysin and other flavonoids in U-251, MDA-MB-231 and PC3 cells.

Other studies have reported the effects of chrysin, including in NSCLC and colon carcinoma. For example, chrysin (and wogonin), have been reported to have potential as adjuvant therapy for drug-resistant NSCLC, especially in patients with AKR1C1/1C2 overexpression [33]. This study evaluated the effect of flavonoids and demonstrated that IL-6-induced AKR1C1/1C2 overexpression and drug resistance can be inhibited by chrysin and wogonin, which both demonstrated multiple anti-inflammatory effects in these cells. Chrysin has also been demonstrated to cause SW480 cells to arrest at the G2/M phase of the cell-cycle in a dose-dependent manner [34]. Combining chrysin with apigenin was found to double the proportion of SW480 cells in G2/M. Thus, apigenin-related flavonoids such as chrysin, may cooperatively protect against colorectal cancer through conjoint blocking of cell-cycle progression. Chrysin also inhibited the lipopolysaccharide-induced COX-2 expression via inhibition of nuclear factor IL-6 (NF-IL6) [45]. Thus, chrysin might also improve the drug sensitivity of cancer cells by modulating the signaling pathways of inflammatory cytokines.

Perhaps the biological activities of chrysin could be improved by combination with other flavonoids, as combinations of flavonoids have been demonstrated to have better apoptotic effects than individual use of chrysin. For example, the combination of chrysin with apigenin, baicalin and scutellarein (5 μM each) inhibited the proliferation of U87-MG glioma cells by almost 50%, while chrysin alone showed no anti-proliferative activity in these cells [11].

Besides, modified chrysin is demonstrated to exhibit more potent anti-cancer effects than the unmodified chrysin. In addition to the inhibitory effects of phosphorylated chrysin in HeLa cells, as mentioned above, 5-allyl-7-gen-difluoromethylenechrysin (ADFMChR) has shown to inhibit the proliferation of human ovarian cancer cells, CoC1, in a dose-dependent manner (IC_50_ of 7.76 μmol/L) [46]. The ADFMChR significantly induced apoptosis in this cell line in a concentration-dependent manner, with rates of apoptosis of 33.07% and 73.70% after the cells were treated with 10.0 and 30.0 μmol/L of ADFMChR, respectively, for 48 h. The apoptosis rate was compared with the cells treated with 10.0 and 30.0 μmol/L of unmodified chrysin, which rates the apoptosis of 21.70% and 40.00%, respectively. Moreover, another study investigating the effects of 5,7-dihydroxy-8-nitrochrysin (NOChR) on apoptosis in human gastric carcinoma cell line, SGC-7901, showed that NOChR markedly inhibited the proliferation of SGC-7901 cells in a dose-dependent manner, where the potency of NOChR (IC_50_ of 4.14 μmol/L) was 10 times higher than that of unmodified chrysin (IC_50_ of 40.56-μmol/L) [47]. Overall, all these studies suggest that modified chrysin could exhibit more potent anti-cancer effects than the unmodified chrysin.

It is also possible that the potency of chrysin is improved by addition of more hydroxyl constituents. According to Monasterio *et al*. [26], at least two hydroxyls at positions 3, 5 or 7 of the A ring were needed to confer the pro-apoptotic activity. This phenomenon can be seen in 4′,7-dihydroxyflavone, chrysin and galangin where the 4′,7-dihydroxyflavone has only one hydroxyl at position 7 of the A ring did not show pro-apoptotic activity, whereas the chrysin (which has two hydroxyls at positions 5 and 7 of the A ring) and galangin (which has three hydroxyls at positions 3, 5 and 7 of the A ring) have higher pro-apoptotic activity, in accordance. Moreover, the hydroxyls at 3′ and/or 4′ positions of the B ring are to increase the pro-apoptotic activity of the flavanoids, according to the study. However, the number of hydroxyl constituents in the B ring is not a good marker of the potential pro-apoptotic activity. Flavanoids that are not hydroxylated in the B ring, such as chrysin and galangin, are potent inducers of apoptosis. Introduction of hydroxyls may also lead to disturbance of the structure of flavanoids.

## 6. Conclusions

Chrysin inhibits proliferation and induces apoptosis in most cancer cells tested, and is likely more potent than other flavonoids in leukemia cells. Studies of the mechanism of action suggest that the chrysin is likely to act via caspase activation and inactivation of the Akt signaling. The biological activities of chrysin, perhaps, may be improved by combination with other flavonoids and modifications to the structure of chrysin. Although most studies support the conclusion that chrysin induces apoptosis in various tumor cell lines, the mechanism of induction of apoptosis remains unclear. Studies published so far are often haphazard and sometimes contradictory. Therefore, more studies are warranted to identify the potential molecule target of chrysin involved in the modulation of apoptosis in human cancer *in vitro*.

## Figures and Tables

**Table 1.** The apoptotic effects of chrysin in human cervical cancer, leukemia, esophageal squamous carcinoma, malignant glioma, breast carcinoma and prostate cancer *in vitro*.

**Cancer type**	**Reference**	**Effect and molecular mechanism**
**Cervical cancer**	[24]	Chrysin (IC_50_ = 14.2 μM) inhibited proliferation and induced apoptosis in HeLa cells, though the effects were not as potent as those of its synthetic derivative compounds.
	[25]	Chrysin (30 μM) potentially induced p38 and NFkappaB/p65 activation in HeLa cells.
**Leukemia**	[26]	Chrysin (IC_50_ = 16 μM) showed to be the most potent flavonoid to reduce cell viability and induce apoptotic DNA fragmentation in U937 cells.
	[27,28]	Chrysin induced apoptosis in Bcl-2 overexpressing U937 leukemia cells, was associated with activation of caspase-3 and PLC-γ1 degradation. The induction of apoptosis was accompanied by down-regulation of XIAP and inactivation of Akt.
	[29]	Chrysin had the ability to abolish SCF/c-Kit signaling by inhibiting the PI3K pathway in MO7e, myeloid leukemia cells.
	[30]	Chrysin, alone or in combination with other compounds, decreased Akt phosphorylation and potentially caused mitochondrial dysfunction in THP-1 and HL-60 leukemia cells.
**Esophageal squamous carcinoma**	[31,32]	Chrysin (IC_50_ = 63 μM) induced cytotoxicity in KYSE-510 cells in dose- and time-dependent manners.
**Malignant glioma, breast carcinoma, prostate cancer**	[11]	Chrysin (100 μM) showed dose-dependent inhibition of U87-MG, MDA-MB-231, U-251 and PC3 proliferation, and displayed apoptotic activity in U87-MG cells. However, the study did not report details about the apoptotic activity of chrysin in U-251, MDA-MB-231 and PC3 cells.
**NSCLC**	[33]	Chrysin and wogonin showed to have potential as adjuvant therapy for drug-resistant NSCLC, especially in patients with AKR1C1/1C2 overexpression where IL-6-induced AKR1C1/1C2 overexpression and drug resistance could be inhibited by these flavonoids in H23 cells.
**Colon cancer**	[34]	Chrysin caused the SW480 cells in cell-cycle arrest at the G2/M phase in a dose-dependent manner.
